# X-Ray Scatter Correction on Soft Tissue Images for Portable Cone Beam CT

**DOI:** 10.1155/2016/3262795

**Published:** 2016-02-16

**Authors:** Sorapong Aootaphao, Saowapak S. Thongvigitmanee, Jartuwat Rajruangrabin, Chalinee Thanasupsombat, Tanapon Srivongsa, Pairash Thajchayapong

**Affiliations:** ^1^X-Ray CT and Medical Imaging Laboratory, National Electronics and Computer Technology Center, National Science and Technology Development Agency, 112 Thailand Science Park, Phahonyothin Road, Khlong Nueng, Khlong Luang, Pathum Thani 12120, Thailand; ^2^National Science and Technology Development Agency, 111 Thailand Science Park, Phahonyothin Road, Khlong Nueng, Khlong Luang, Pathum Thani 12120, Thailand

## Abstract

Soft tissue images from portable cone beam computed tomography (CBCT) scanners can be used for diagnosis and detection of tumor, cancer, intracerebral hemorrhage, and so forth. Due to large field of view, X-ray scattering which is the main cause of artifacts degrades image quality, such as cupping artifacts, CT number inaccuracy, and low contrast, especially on soft tissue images. In this work, we propose the X-ray scatter correction method for improving soft tissue images. The X-ray scatter correction scheme to estimate X-ray scatter signals is based on the deconvolution technique using the maximum likelihood estimation maximization (MLEM) method. The scatter kernels are obtained by simulating the PMMA sheet on the Monte Carlo simulation (MCS) software. In the experiment, we used the QRM phantom to quantitatively compare with fan-beam CT (FBCT) data in terms of CT number values, contrast to noise ratio, cupping artifacts, and low contrast detectability. Moreover, the PH3 angiography phantom was also used to mimic human soft tissues in the brain. The reconstructed images with our proposed scatter correction show significant improvement on image quality. Thus the proposed scatter correction technique has high potential to detect soft tissues in the brain.

## 1. Introduction

Cone beam computed tomography (CBCT) scanners have been initially developed for dental applications. Dental CBCT images usually represent only bone structures due to limited contrast resolution of X-ray detectors. In recent years, flat panel detectors (FPDs) were tremendously improved in their capability of detecting small differences in attenuation of X-ray beam, thus providing soft tissue detectability, such as tumor, muscle, and intracerebral hemorrhage (ICH) [1–4]. This particular FPD model has been introduced in portable CBCT scanners with large field of view (FOV) to cover the head region. The portable CBCT scanner can be freely moved where rapid diagnosis is needed such as in the emergency and operation rooms [2–4]. The scanner can perform patient screening before treatment, during operation, and after operation. For having a large FPD, soft tissue images from CBCT scanner are usually degraded as more artifacts occur such as lag, glare [5], motion artifact [4, 5], beam hardening effects (BHE) [4–6], and X-ray scattering effects [5–7]. However, it is well known that the most critical artifact is caused by the X-ray scattering effect which reduces image quality. X-ray scatter signals directly affect contrast and CT numbers of soft tissue images. The number of X-ray scatter signals is increased as an increase in FOV and object thickness [7].

There are many methods to reduce X-ray scatter signals in FPDs, such as antiscatter grid plate [8], beam stop array plate [9], primary modulation methods [10], Monte Carlo simulation (MCS) software [11, 12], and X-ray scattering models [13–18]. The antiscatter grid plate can reduce X-ray scatter signals to increase image quality; however, it is not sufficient for improving image quality on soft tissue images [8], and the apparent dose increase is always undesired. A beam stop array plate [9] can be used for measuring X-ray scatter signals; however, this method is not practical in clinics. The primary modulation method was to place a high frequency attenuation plate between an X-ray source and an object to obtain a modulated projection image, and then this modulated projection image was filtered to eliminate low-frequency components due to X-ray scatter signals by a high-pass filter [10]. Monte Carlo simulation software based on Geant4 libraries [11, 12] has been used widely to design the simulated system for estimating high accuracy X-ray scatter signals. Generally, the MCS software usually utilized expensive computation time. The technique proposed by Star-Lack et al. [14] described efficient X-ray scattering correction on the large ellipse phantom using asymmetric kernels. Since our work aims to reduce the X-ray scatter signals in the patient's head only, symmetric kernels can be assumed. Several iterative techniques were proposed for scatter correction including subtraction from measured data [14, 15] and deconvolution by the statistical method [13, 16, 17]. The problem of the subtraction method is that the corrected value can become negative as discussed in [16].

In this work, we propose the X-ray scattering correction method for improving soft tissue images on the large flat panel detector of portable CBCT. We apply the deconvolution technique based on the maximum likelihood expectation maximization (MLEM) method [13, 16, 17] for estimating the primary signal. First, the MCS software is used to form the shape and the amplitude of the X-ray scatter signals as a point spread function (PSF) and a scatter fraction (SF) function according to actual parameters of the CBCT system. Second, we match equivalent thickness in projection images to obtain subprojection images. Third, those subprojection images are convolved with the prepared kernels or PSFs and combined to obtain a convolved projection image. Finally, the convolved projection image is used in the MLEM method to estimate the primary signal, and the process is iterated until convergence to FBCT data. Once the primary signal is estimated, the reconstruction algorithm based on filtered backprojection (FBP) technique [19] is employed. In the experiment, our proposed technique will be tested with the QRM-ConeBeam phantom by QRM GmbH, Germany [20], and the PH3 angiographic CT head phantom ACS by Kyoto Kagaku Co., Ltd., Japan [21]. The experimental results are compared with FBCT data.

## 2. Materials and Methods

### 2.1. Materials

**Figure 1:**
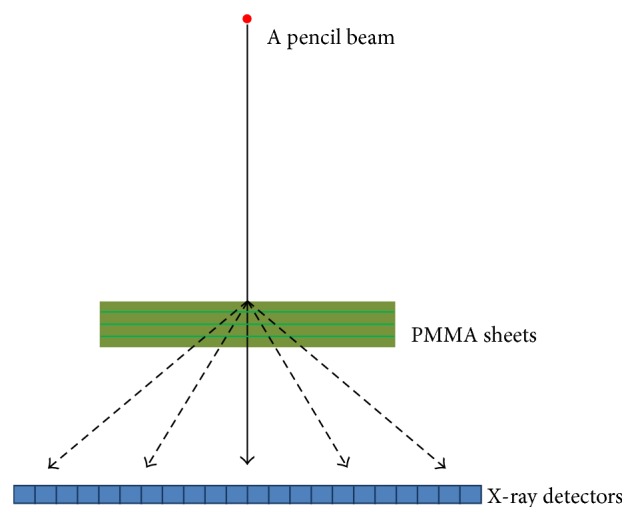
The X-ray scatter signal measurement model in the Monte Carlo simulation software.

Our prototype portable CBCT scanner prototype consists of the flat panel detector (Varian PaxScan 4030CB) and the high frequency X-ray source which shoots X-ray pulses by synchronizing with exposure time of the flat panel detector. The distance from source to detector (DSD) and the distance from source to an object (DSO) are 500 mm and 786 mm, respectively. The dynamic gain was operated for the pixel size of 0.388 mm. This system used 90 kVp, 9 mA, and the total filtration of 0.5 mm Aluminum. A full rotation of scanning was achieved. In this work, we used PSF and SF from MCS software to simulate X-ray scatter signals by using PMMA sheets with a pencil beam as shown in Figure 1.

**Figure 2:**
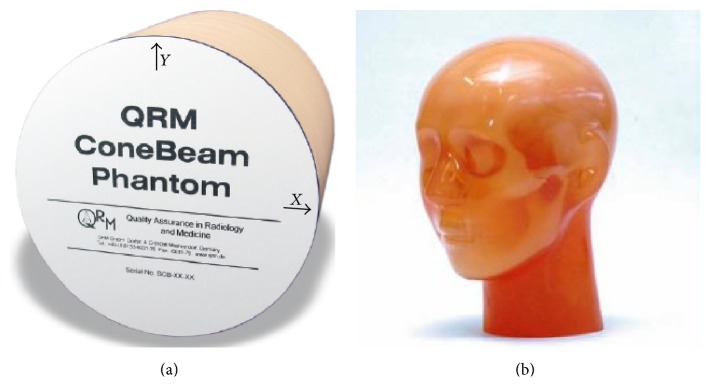
(a) QRM-ConeBeam phantom and (b) PH3 angiography CT head phantom.

The MCS software computed the X-ray scatter signals by simulating a pencil beam with the PMMA sheets; one set of the PMMA sheets was 38 mm thick and the total of 10 sets was used. The measured X-ray scatter signals at each thickness are interpolated to achieve X-ray scatter signals at thickness of 1 mm. We used 2 phantoms in the experiments: the QRM-ConeBeam phantom by QRM GmbH, Germany [20], is a cylindrical tissue equivalent phantom at 120 kVp with the diameter of 160 mm and the height of 160 mm and the PH3 angiographic CT head phantom ACS by Kyoto Kagaku Co., Ltd., Japan [21], is a life-size adult phantom made of Urethane base resin (SZ-50) and Epoxy base resin as shown in Figure 2.

### 2.2. Method

According to the fundamental law of physics, while the X-ray beam travels to penetrate each layer inside an object, there exist three phenomena of interactions: photoelectric effect, Compton scattering, and Rayleigh Scattering of X-ray photons. The Compton scattering effect is essential for cross section images reconstruction. The X-ray beam that penetrates an object and goes directly to the flat panel detector is desired; however, scattered X-rays from other directions are usually combined at the same sensor position of the flat panel detector. This degrades image quality, such as cupping artifacts, low contrast, and inaccurate CT numbers. We can write the measured X-ray signal model as follows:

(1) $$\begin{array}{c} I_{m}\left(\;x,y\;\right)=I_{p}\left(\;x,y\;\right)+I_{s}\left(\;x,y\;\right), \end{array}$$

where *I*_*m*_ is the measured signal, *I*_*p*_ is the primary signal which is the expected signal to be used in cross section image reconstruction, *I*_*s*_ is the scatter signal, and (*x*, *y*) denotes the pixel coordinate in the projection image. The scatter signal can be written in the form of the primary signal convolved with the kernel function, *K*, as follows:

(2) $$\begin{array}{c} I_{s}\left(\;x,y\;\right)=K\left(\;x,y\;\right)**I_{p}\left(\;x,y\;\right), \end{array}$$

where *∗∗* denotes a 2D convolution operator. The statistical method is based on Bayes' rule as discussed in [13]. The average value of the statistical method is considered by the X-ray photons behind an object as Poisson distribution [13]. The maximum likelihood is used to estimate the primary signal, *I*_*p*_, and can be written in the form of the MLEM [13, 16, 17] algorithm as follows:

(3) $$\begin{array}{c} I_{p}^{n+1}\left(\;x,y\;\right)=\frac{I_{p}^{n}\left(x,y\right)I_{m}\left(x,y\right)}{I_{p}^{n}\left(x,y\right)+I_{p}^{n}**K\left(x,y\right)}, \end{array}$$

where *I*_*p*_^*n*^ is the estimated primary signal at the *n*th iteration. The kernel measurement at different thickness, *K*_*t*_, is obtained according to the thickness of the PMMA sheet, so *I*_*p*_^*n*^ is divided according to thickness, *I*_*p*,*t*_^*n*^. We can rewrite the above equation as follows:

(4) $$\begin{array}{c} I_{p}^{n+1}\left(\;x,y\;\right)=\frac{I_{p}^{n}\left(x,y\right)I_{m}\left(x,y\right)}{I_{p}^{n}\left(x,y\right)+\sum_{t}I_{p,t}^{n}**K_{t}\left(x,y\right)}. \end{array}$$

**Figure 3:**
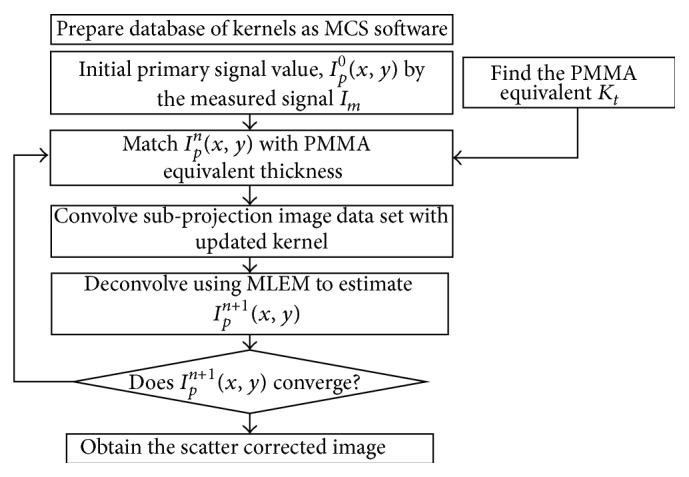
The scatter correction process.

The scatter correction method starts by creating a database of kernels according to thickness of PMMA sheets using the MCS software and initializing the primary signal, *I*_*p*_^0^. The next step is to match each pixel of the primary signal with the estimated PMMA equivalent thickness; that is, the projection image data are divided into different groups or subprojection image data according to thickness. The subprojection image data sets of the primary signal are convolved with the kernels and summed up to obtain the convolved projection image. Then the convolved projection image is used to perform deconvolution by the MLEM algorithm. Finally, we check the condition for convergence. If it does not converge, we will return to the thickness mapping process again. In summary, all steps of the scatter correction can be illustrated as in Figure 3.

#### 2.2.1. Kernel Measurements

**Figure 4:**
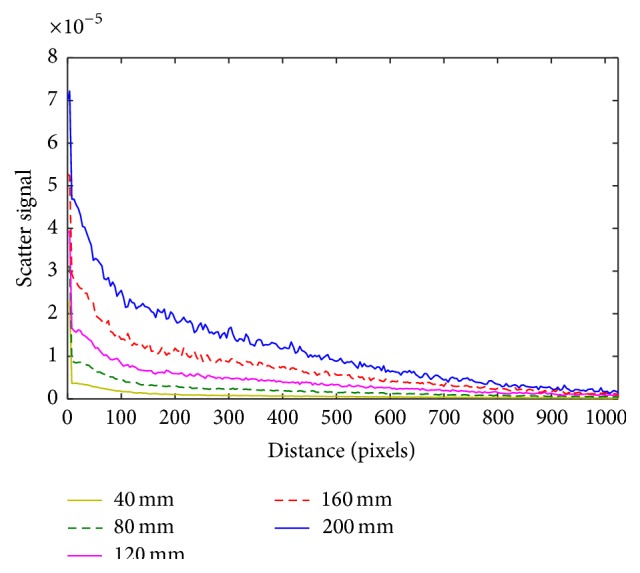
Profiles of scatter signals at each thickness of PMMA sheet.

Kernel measurements in this work are obtained by measuring different thickness of the object. Generally, kernels can be measured from experiments or simulated by the MCS software. For accuracy of measuring the X-ray scatter signal, we used the MCS software to simulate the scatter signals according to the actual CBCT scanner system: 90 kVp Voltage as a pencil beam and the total flat filtration of 5.5 mm Al. The pencil beam penetrates through PMMA sheets. We used the total of 10 sets of PMMA sheets with the thickness of 38 mm each set. The measured scatter signals are interpolated to obtain the scatter signal at each 1 mm thickness, so the total of 380 scatter signals is obtained. The example of scatter signals using this work is shown in Figure 4. They are normalized to obtain the point spread function (PSF) according to thickness, PSF_*t*_. The kernel at each thickness can be described as follows:

(5) $$\begin{array}{c} K_{t}\left(\;x,y\;\right)=A\left(\;t\;\right)\text{SF}\left(\;t\;\right){\text{PSF}}_{t}\left(\;x,y\;\right), \end{array}$$

where *x* and *y* denote the spatial coordinate, *t* is PMMA thickness, and *A* is the compensating value from the experiments, which depends on thickness. The values of *A* used in this work are shown in Table 1. Moreover, the scatter fraction values are measured as an average at the center region of each kernel for a suitable size of region of interest (ROI). In the experiments, we employ linear curve fitting to the measured average value of scatter fraction. The scatter fraction, *I*_*s*_/*I*_*m*_, can be described as follows:

(6) $$\begin{array}{c} \text{SF}\left(\;t\;\right)=a_{1}t+a_{2}, \end{array}$$

where *a*_1_ and *a*_2_ are coefficients of the linear function. The SF value depends on object thickness and highly affects the amount of scatter correction.

#### 2.2.2. Thickness Map Measurements

We match the projection image data to obtain the subprojection image data set according to PMMA thickness. First, we create the log signal function from pure PMMA plates for thickness mapping. We divide thickness of the projection image, *t*_PMMA_, by using Beer's law [11, 12, 19] as follows:

(7) $$\begin{array}{c} t_{\text{PMMA}}=\frac{1}{\mu_{\text{PMMA}}}\log\left(\;\frac{I_{p,0}}{I_{p}}\;\right), \end{array}$$

where *μ*_PMMA_ is the linear attenuation coefficient at the effective energy, *I*_*p*,0_ is the measured primary signal without attenuation, and *I*_*p*_ is the primary signal. We divide the projection image data of the object into the subprojection image data set as the log signal function of PMMA sheet thickness. The measured primary signal *I*_*p*_ at different PMMA thickness from the MCS software can create the log signal function as the relationship between actual PMMA thickness and the log signal value. This function is used for transferring the log signal value to the equivalent value of PMMA thickness.

#### 2.2.3. Evaluation

Evaluation in the experimental results starts as checking the performance of the MLEM method whether it converges according to the log likelihood function, *L* [13]. For simplicity, we ignore the insignificant terms as follows:

(8) $$\begin{array}{c} L=\sum_{x,y}\left[\;I_{m}\left(\;x,y\;\right)\log\left(\;I_{p}^{n}\left(\;x,y\;\right)\;\right)-I_{p}^{n}\left(\;x,y\;\right)\;\right]. \end{array}$$

**Figure 5:**
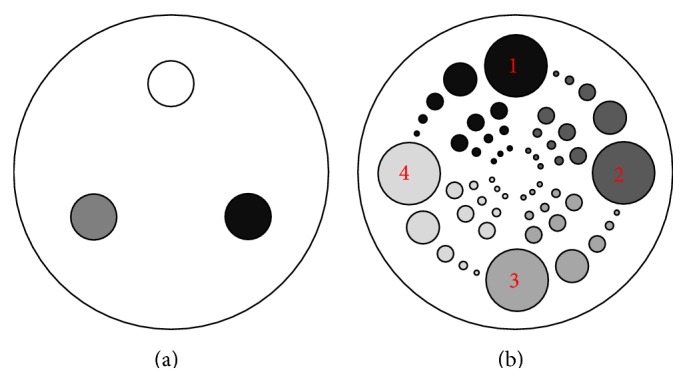
Sections in the QRM-ConeBeam phantom: (a) CT number section and (b) the pattern of low contrast section in Sections A, B, and C.

To verify our proposed algorithm, we will compare both projection images and cross section images with the FBCT data. The CT number in the reconstructed images is calibrated by using the water average value in the CT number section of the QRM-ConeBeam phantom as shown in Figure 5(a). Note that the water average value is measured by using FBCT instead of CBCT to avoid scatter effects. We can normalize the Hounsfield unit (HU) as follows:

(9) $$\begin{array}{c} \text{CT}\#=1000\times \frac{\left(m_{x}-m_{\text{water,FBCT}}\right)}{m_{\text{water,FBCT}}}, \end{array}$$

where *m*_*x*_ is the average value of any material in the cross section images and *m*_water,FBCT_ is the water average value from FBCT. To measure the low contrast detectability, we use Sections A, B, and C of the QRM-ConeBeam phantom. Section A has higher contrast between inserts and background than Sections B and C, while Section C has the lowest contrast. Here we measure the density value at numbers 1 to 4 as shown in Figure 5(b). Different gray level values represent different density values.

After estimating scatter correction, the contrast is increased; however, the noise signal value in the projection images is increased as well. In this study, we measure the contrast value between two different inserts and its contrast to noise ratio (CNR) as follows:

(10) Contrast=mx−mbackground,CNR=mx−mbackgroundσx2+σbackground2,

where *m*_background_ is the average value in the background region and *σ*_*x*_ and *σ*_background_ are the standard deviation (STD) value of any insert material and the background, respectively. However, we also measure percentage of cupping in the cross section images in order to evaluate the proposed method and the remaining beam hardening effect as follows:

(11) $$\begin{array}{c} \%\text{ cupping}=\frac{\left({\text{CT}\#}_{\text{edge}}-{\text{CT}\#}_{\text{center}}\right)\times 100}{\left({\text{CT}\#}_{\text{edge}}+1000\right)}, \end{array}$$

where CT#_edge_ is an average CT number value from four peripheral ROIs in the uniform areas and CT#_center_ is the average CT number value at the center.

## 3. Results

All parameters used in this study are shown in Tables 1 and 2.  Table 2 shows the implementation parameters including the coefficient values of the SF function: *a*_1_ = 0.0038, *a*_2_ = 0.1; number of groups: 5; and the thickness of each group: 40 mm. From the experiment, we tried dividing into different groups and found that five groups with 40 mm thickness in each group were sufficient for acceptable estimation of the primary signal. Moreover, according to the experiment, the proposed method did not introduce any additional artifacts.

### 3.1. Scatter Correction Results in the Projection Image

**Figure 6:**
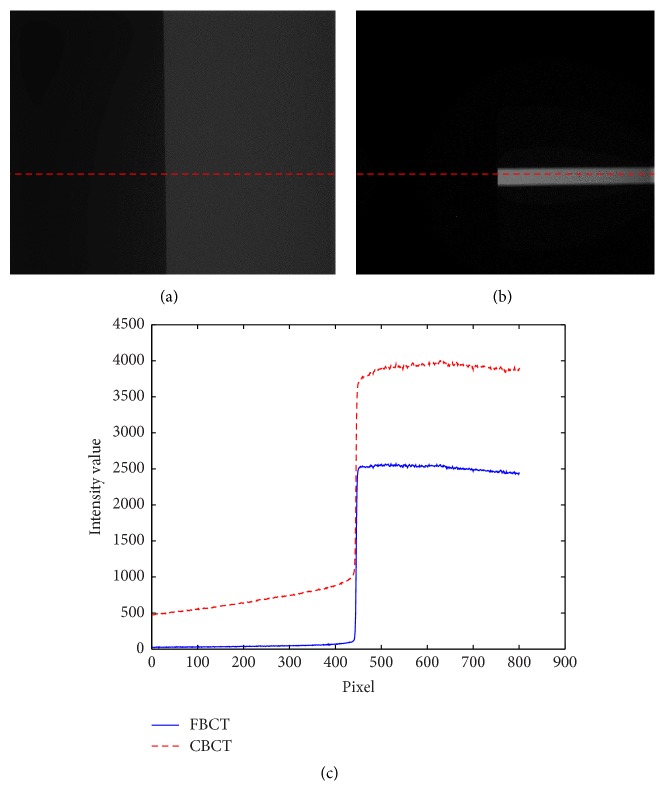
(a) The projection image from CBCT, (b) the projection image from FBCT, and (c) CBCT and FBCT profile data comparison.

The scatter correction results in the projection images were compared with the FBCT data. FBCT was obtained from the narrow-collimated scan of the CBCT system. The collimation was made of 3 mm thick leaded blades with 3 mm opening in the vertical direction. Due to narrow collimation, the FBCT profile data contain less scatter signal than the profile data obtained from CBCT. In the experiment, we used the PMMA sheet (thickness of 60 mm) that is attached with the lead sheet (thickness of 3 mm) for measuring the scatter signal value and comparing its profile with FBCT as shown in Figure 6.

**Figure 7:**
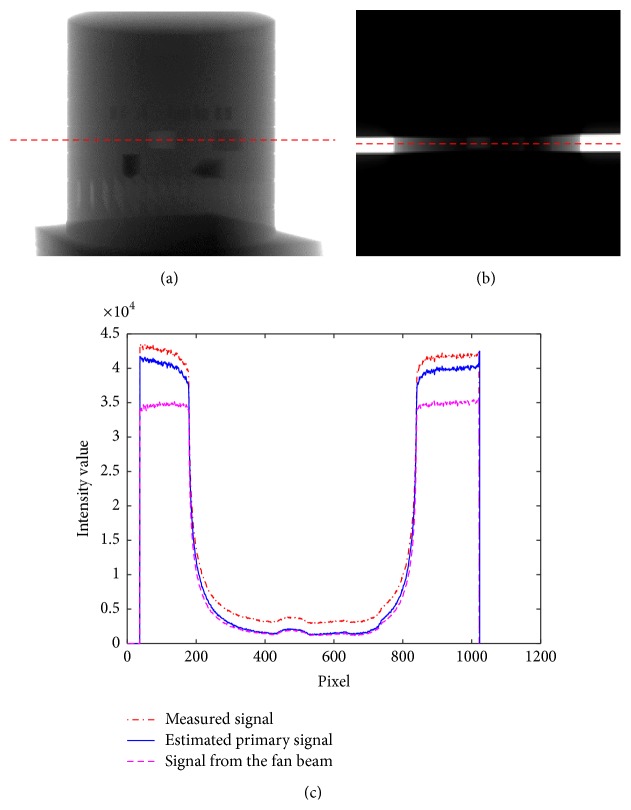
(a) The projection image of QRM-ConeBeam phantom in CBCT, (b) the projection image of the QRM-ConeBeam phantom in FBCT, and (c) comparison of profiles data before and after scatter correction with FBCT data.

**Figure 8:**
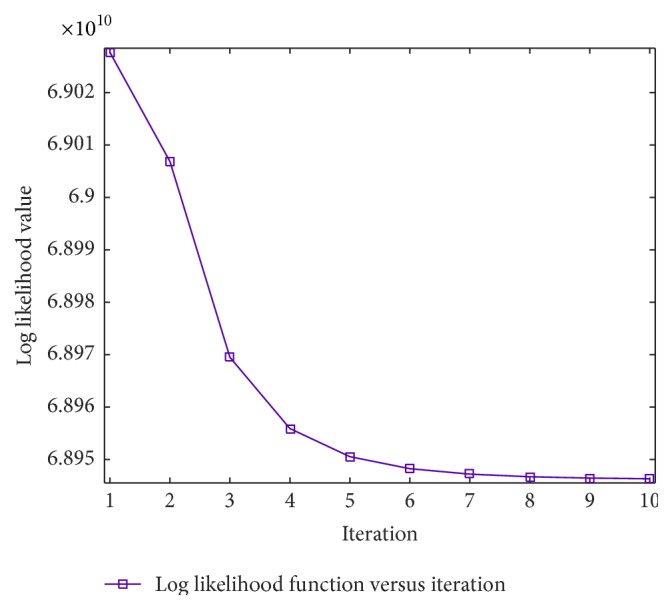
Log likelihood value versus number of iterations.

Ideally, the profile data obtained from FBCT should be almost identical to the actual primary signal; therefore, the FBCT data are used as the benchmark in comparison with the estimated data from CBCT. However, FBCT acquisition is limited to only a small strip around the center of the X-ray beam.  Figure 7 shows comparison among uncorrected CBCT and corrected CBCT and FBCT profiles of the projection data acquired from the QRM-ConeBeam phantom. The log likelihood values calculated using (8) are plotted versus the number of iterations as shown in Figure 8. The log likelihood values seem to converge as the number of iterations increases. One of the stopping criteria can be monitored from the convergence of the log likelihood values. After the convergence of the log likelihood value, the reconstructed cross section images of the proposed scatter correction method should be close to the cross section image of the FBCT.

### 3.2. Measurements in the CT Number Section

**Figure 9:**
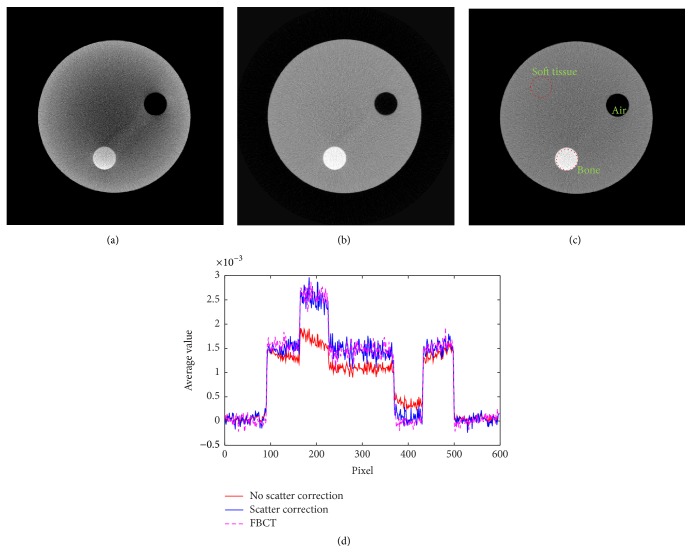
(a) Reconstructed image without scatter correction, (b) with scatter correction, (c) FBCT, and (d) comparison of profile data with and without scatter correction and FBCT data.

Corrected projection images are reconstructed by the filtered backprojection method [19] using the Shepp Logan filter with the cutoff at 0.6 and the voxel size of 0.3 mm. The cross section images in the CT number section are measured and compared with FBCT data.  Figure 9 shows the cross section images with and without scatter correction and their profiles comparison. All reconstructed images in Figure 9 are displayed with the window width and level (*W*/*L*) of 1500 and 1000, respectively.

The profile data comparison is plotted across the bone insert and the air hole in the CT number section as shown in Figure 9(d). The proposed profile is almost identical to the profile from FBCT as the cupping effect is reduced. The CT number values are calculated by using the average value of the water insert according to the QRM-ConeBeam phantom's specifications [20]. Since the X-ray spectrum we used cannot clearly discriminate the density value of water and soft tissue (background), we used the soft tissue value instead. In the experiment, we measured the CT numbers of three inserts up to 10 iterations as shown in Table 3.

The accuracy of the CT number value at each insert increases as the number of iterations increases; however, once convergence is reached the accuracy of CT number value stays the same even when we perform more number of iterations. These CT number values are considered to be valid for diagnosis after convergence is achieved. However, noise is also increased after scatter correction. We evaluated the contrast value between bone and soft tissue inserts and the influence of noise by measuring the contrast to noise ratio as shown in Table 4. In Table 5, to calculate percentage of cupping, we compare the center average value with the average value of four peripheral areas using (11).

From Table 5, the cupping artifacts are significantly reduced, comparing with FBCT data; however, the small effect from beam hardening is still present even with FBCT. As the log likelihood value is decreased, the CT number values of bone, air, and soft tissue as well as the contrast values are significantly improved; however, noise is also increased. In this study, we did not perform noise suppression after scatter correction, so this would affect the CNR.

### 3.3. Measurement Results in the Low Contrast Sections and the PH3 Phantom

**Figure 10:**
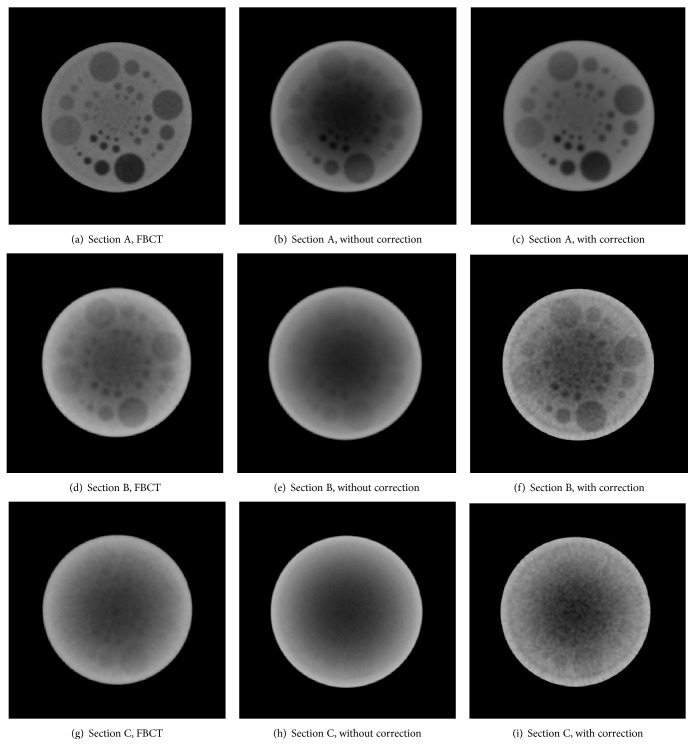
Result comparison between CBCT with and without scatter correction and FBCT in each section of low contrast detectability (window/level: 500/−50).

There are three sections of low contrast detectability in the QRM-ConeBeam phantom, namely, Sections A, B, and C. They have the same pattern as shown in Figure 5(b), but the density values of the inserts within each section are different. The results of three low contrast sections after 5 iterations of scatter correction are reconstructed by the FBP method using the Hamming filter with the cutoff frequency of 0.6 and the voxel size of 0.6 mm and then compared with FBCT as shown in Figure 10. We measured the CT numbers in each section according to numbers 1 to 4 as shown in Figure 5(b) and the errors were calculated by using the absolute different value of the corrected results with FBCT.

The measured HU values in three sections of low contrast are shown in Tables 6 [Table tab7]–8. Note that one of the reasons that the error after scatter correction in Section A seems to be a little higher than Sections B and C might be because the position of Section A is further away from the central ray than Sections B and C.

**Figure 11:**
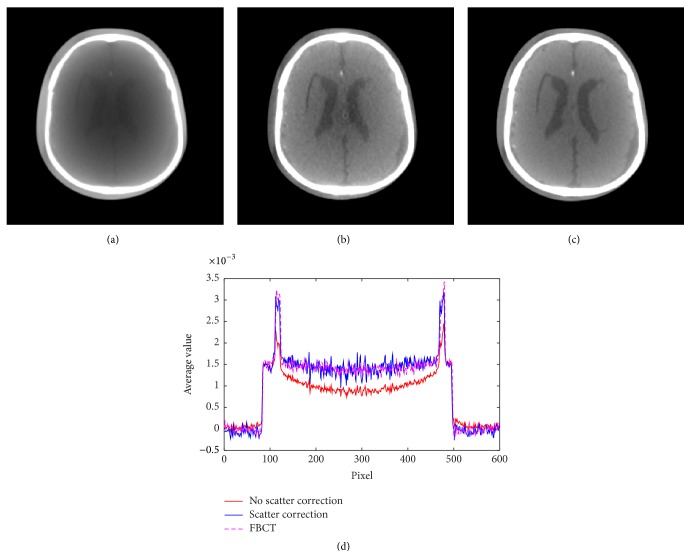
(a) CBCT without correction, (b) CBCT with correction, (c) FBCT, and (d) profile comparison.

**Figure 12:**
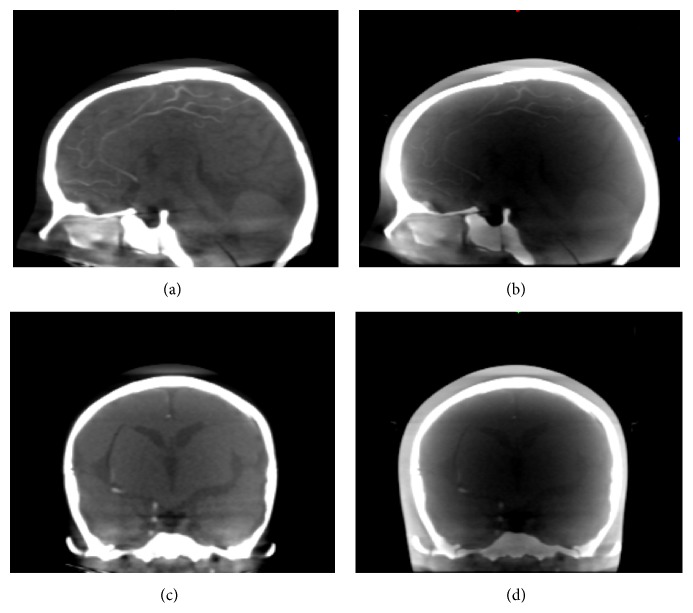
(a) The sagittal plane with correction, (b) the sagittal plane without correction, (c) the coronal plane with correction, and (d) the coronal plane without correction.

Results in three sections of low contrast indicate better image quality. The low contrast values in three sections can be discriminated; thus the contrast is significantly improved. Moreover, some inserts appear after correction. In addition to the QRM-ConeBeam phantom, we applied the proposed method to another phantom using the same parameters and the results are shown in Figures 11 and 12. The reconstructed images in these two figures are displayed with the window width and level of 900 and −100, respectively.  Figure 11 shows the cross section images of the PH3 phantom along with profile data comparison. The ventricles region in the PH3 angiographic CT head phantom is significantly improved as shown in Figure 11(b). The corrected profile in Figure 11(d) is very close to the FBCT profile.  Figure 12 shows the reconstructed images with and without scatter correction in the coronal and sagittal planes. Although image quality is improved, the beam hardening effect still presents in these planes.

## 4. Discussion

Although the log likelihood function seems to converge as the number of iterations increases, image quality after scatter correction may not be approaching FBCT exactly according to the log likelihood function. The small change in the log likelihood value after several iterations may result in overcorrection in the reconstructed images as indicated by the percentage of cupping in Table 5. For example, the percentage of cupping at 10 iterations is smaller than that of FBCT. Therefore, to ensure convergence and avoid overcorrection, only 5 iterations seem to be sufficient (Figures 10–12). In this work, we attempt to improve image quality of low contrast by applying the proposed method to the three sections of low contrast in the QRM-ConeBeam phantom. The results of three sections in Figure 10 are significantly improved; that is, visibility of some inserts in Section C can be observed. Moreover, in Table 5, the percentage of cupping artifacts in FBCT is about 10% which means that other causes of cupping artifacts besides scattering are present. One of them could be beam hardening effects, which is ignored in this study.

## 5. Conclusions

In this paper, we propose the scatter correction method to improve image quality of soft tissue images acquired from portable CBCT. Our proposed technique is based on estimation of X-ray scatter signals using the MLEM method and kernel modeling with Monte Carlo simulation. By benchmarking with FBCT, the scatter correction results with CBCT show significant improvement on image quality. With the QRM-ConeBeam phantom, the cupping artifacts are reduced, CT numbers of inserts are approaching FBCT, contrast is increased, and low contrast detectability becomes more apparent. Moreover, scatter correction in the reconstructed images of the PH3 angiographic CT head phantom can bring out soft tissue structures prominently. Therefore, our proposed scatter correction method has a high possibility to detect soft tissue images using portable CBCT. For future work, we will test our proposed technique on real patient data.

## Figures and Tables

**Table 1:** Compensating value of *A* in each thickness range.

	0 < *t* ≤ 40 mm	40 < *t* ≤ 80 mm	80 < *t* ≤ 120 mm	120 < *t* ≤ 160 mm	160 mm < *t*
*A*	1.0	1.0	1.75	1.75	2.0

**Table 2:** Parameter setting for the proposed scatter correction method.

SF(*t*) = *a*_1_ · *t* + *a*_2_	Thickness of each group	Number of groups
*a*_1_ = 0.0038, *a*_2_ = 0.1	40 mm	5

**Table 3:** CT number value comparison in the QRM-ConeBeam phantom.

	FBCT		No correction		Scatter correction at different iterations									
					2 iterations		4 iterations		6 iterations		8 iterations		10 iterations	
	HU	STD	HU	STD	HU	STD	HU	STD	HU	STD	HU	STD	HU	STD
Bone	752	122	141	82	436	105	648	128	713	139	736	145	742	148
Air	−1000	63	−760	57	−809	76	−905	77	−923	70	−940	67	−943	66
Soft tissue	0	112	−197	63	−60	85	−2	108	6	117	7	120	7	121

**Table 4:** Contrast and CNR values between bone and soft tissue.

	FBCT	No correction	Scatter correction at different iterations				
			2 iterations	4 iterations	6 iterations	8 iterations	10 iterations
Contrast	752	338	496	650	707	729	735
CNR	5.10	3.38	3.72	3.93	3.97	3.96	3.95

**Table 5:** Percentage of cupping artifacts.

	FBCT	No correction	Scatter correction at different iterations				
			2 iterations	4 iterations	6 iterations	8 iterations	10 iterations
% cupping	10	28.83	19.57	10.34	7.54	6.68	6.40

**Table 6:** HU values of different inserts in Section A of the QRM-ConeBeam phantom.

	FBCT	CBCT without correction		CBCT with scatter correction	
	Mean (HU)	Mean (HU)	Different value (HU)	Mean (HU)	Different value (HU)
A1	−165	−269	104	−187	22
A2	−91	−223	132	−112	21
A3	−69	−210	141	−90	21
A4	−45	−195	150	−68	23

**Table 7 tab7:** HU values of different inserts in Section B of the QRM-ConeBeam phantom.

	FBCT	CBCT without correction		CBCT with scatter correction	
	Mean (HU)	Mean (HU)	Different value (HU)	Mean (HU)	Different value (HU)
B1	−8	−164	156	−6	2
B2	7	−156	149	8	1
B3	11	−154	143	12	1
B4	16	−148	132	17	1

**Table 8:** HU values of different inserts in Section C of the QRM-ConeBeam phantom.

	FBCT	CBCT without correction		CBCT with scatter correction	
	Mean (HU)	Mean (HU)	Different value (HU)	Mean (HU)	Different value (HU)
C1	17	−156	139	18	1
C2	21	−152	131	22	1
